# Raman Spectroscopy at Metal Interfaces: A Numerical Study of the Strong Coupling Regime

**DOI:** 10.1021/acs.jpcc.6c03454

**Published:** 2026-09-29

**Authors:** Zeyu Zhou, Maxim Sukharev, Abraham Nitzan, Joseph E. Subotnik

**Affiliations:** † Department of Chemistry, 6740 Princeton University, Princeton, New Jersey 08544, United States; ‡ Department of Physics, Arizona State University, Tempe, Arizona 85287, United States; § College of Integrative Sciences and Arts, Arizona State University, Mesa, Arizona 85212, United States; ∥ Department of Chemistry, 6572 University of Pennsylvania, 231 South 34th Street, Philadelphia, Pennsylvania 19104, United States; ⊥ School of Chemistry, The Raymond and Beverly Sackler Faculty of Exact Sciences and The Sackler Center for Computational Molecular and Materials Science, Tel Aviv University, Tel Aviv 6997801, Israel

## Abstract

We investigate how proximity to a metal nanostructure, particularly to a flat mirror or a cavity confined between two mirrors, affects the vibronic structure of the Raman scattering signals. We find that such proximity, particularly for the strong-coupling situation encountered in cavity environments, plays multiple roles in shaping Raman signals beyond the now familiar signal enhancement known as surface-enhanced Raman scattering (SERS). First, in analogy to the electromagnetic SERS mechanism, near or between mirrors, the local field experienced by a molecule differs from that in vacuum. In particular, between mirrors, the cavity enhances the effective excited-state population by trapping the electromagnetic (EM) field inside it. Second, the nearby metal surface provides a relaxation channel and a lineshape broadening mechanism, and inside a cavity, this lineshape is inherited by the cavity polaritons. This relaxation results in a loss of yield, but the associated broadening also leads to significant absorption over a larger frequency range. Third, near-metallic interface interference between incident and reflected light can lead to a richly structured Raman spectrum. For instance, we find that the Rabi contraction (which results from depopulating the ground state) can interfere with Raman signals (and the effect appears to be of the same order as Raman itself). These cavity effects are calculated by a full-scale finite-difference time-domain (FDTD) simulation and highlight the convoluted but fascinating roles of photonic materials on optical signals.

## Graphical abstract

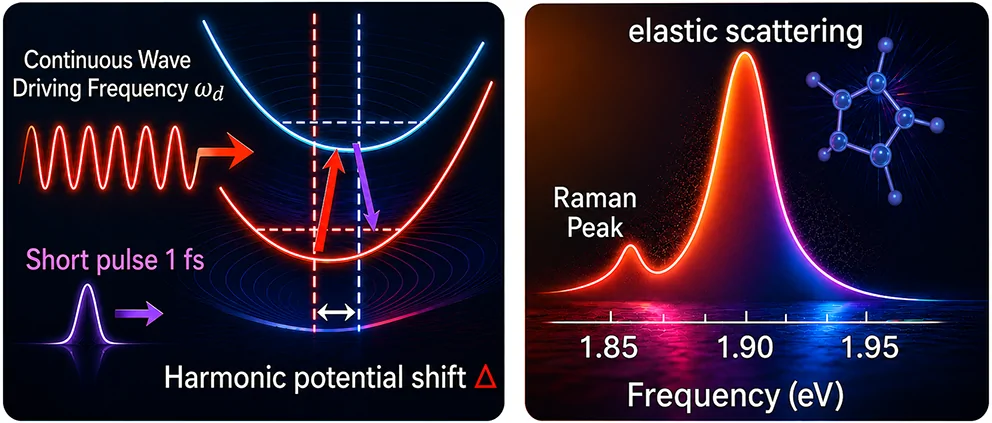

## I Introduction

The enhancement of light–matter interactions near metal and semiconductor interfaces has been a central research area for the last few decades. Scientists possess unprecedented tools with the development of nanotechnologies, which are often based on metallic/composite dielectric nanolayers and nanoparticles. For example, Raman-active molecules near metallic surfaces enable surface-enhanced Raman spectroscopy (SERS), magnifying signals with rich vibrational signatures.^1–6^ Another fruitful area of research involves embedding materials in between two mirrors, leading to the formation of hybrid light–matter states within such nanostructures.^7–21^ The latter phenomena arise from enhanced effective coupling, driven by prolonged cavity photon lifetimes and reduced modal volumes, and have led to many opportunities to engineering compact, highly responsive, and high signal-to-noise ratio nano-optical devices with tailored functionalities for sensing, spectroscopy, and energy harvesting. Because these devices are on the nanometric scale by design, a proper analysis of their capabilities requires a comprehensive semiclassical method that can capture essential quantum effects and macroscopic electrodynamical interactions as applied to heterogeneous optical structures.

To this end, simulations combining classical electrodynamics—implemented through finite-difference time-domain (FDTD) methods—with semiclassical mean-field descriptions of molecular subsystems have proven remarkably successful. Such mixed quantum–classical approaches have demonstrated the ability to reproduce a wide range of strong light–matter phenomena.^22–33^ A major advantage of FDTD lies in its straightforward extraction of optical response functions, which is essential for spectroscopic analysis. Moreover, nonlinear processes involving multiple relevant frequencies can be naturally captured without the prohibitive cost of full quantum simulations.^28,34^ Finally, for dense molecular layers, dipole–dipole interactions become significant and can give rise to pronounced collective behaviors.^34–36^

Of course, a classical description of the radiation field cannot account for the quantum character of spontaneous emission and spontaneous Raman scattering (see, e.g., refs 37 and 38). Moreover, Raman signals are second-order effects, while a strong driving leads to a strong linear background response; this combination of effects has hitherto precluded us from observing clean spontaneous Raman signals. Building on these considerations, the present work investigates the intensity and structure of *stimulated* Raman signals from a molecular layer, comparing the stimulated Raman response of such a layer in vacuum to the analogous response near a single metallic mirror and inside a cavity made of two metallic mirrors. The stimulated Raman signals are obtained by pumping the system with a central driving frequency ω_*d*_ and triggering stimulated emission with a probe short pulse. We obtain the linear absorption and stimulated Raman spectra for molecular models characterized by their free-space Franck–Condon structures, making it possible to examine the effects of the mirror and cavity environment on these structures. Our simulations reveal four key findings. (i) A cavity consisting of two mirrors further enhances Raman signals as compared to a single flat metal surface by promoting a larger absorption cross section. (ii) The formation of cavity polaritons allows more absorption at different energetic regimes. (iii) A low-quality cavity introduces extra broadening on the polaritons, leading to richer Raman signatures over a wider range of frequencies. (iv) The local inhomogeneous dielectric environment can modify the Raman signal profile and exhibit a non-Gaussian/Lorentzian lineshape.

This paper is arranged as follows. In Section 2, we present our model scenarios with one layer of 2-level molecules and one mirror/one cavity, and discuss how we apply the FDTD method and solve the coupled Maxwell–Schrodinger equation to extract stimulated Raman signals. In Section 3, we investigate the absorption and stimulated Raman spectra for molecular models characterized by their free-space Franck–Condon structures. The effect of the driving frequency on the calculated stimulated Raman spectrum is also studied. We conclude in Section 4, where we summarize our findings and discuss the importance of the local dielectric environment on the observed spectra.

## II Methods

### II.I Structure of the Systems

In this work, we consider three scenarios. First, as shown in Figure 1(a), we imagine a single layer of molecules in vacuum. Second, as shown in Figure 1(b), we add a thick silver mirror (70 nm) on the right-hand side of the same molecular layer as in Figure 1(a). Last, as shown in Figure 1(c), we place a thin silver mirror (20 nm) on the left side of the molecular layer, and thus a relatively low-quality cavity is formed. Note that the stimulated Raman signal is often off-resonance with the cavity polaritons and therefore relatively weak. The use of a thin mirror ensures large enough Raman signals to leak out and get collected on the left side. In Figure 1(d), we sketch the relevant molecular energy diagram. In our numerical implementation, each spatial grid point represents a molecule, modeled by one harmonic vibrational mode {|0⟩, |1⟩, ...} (ℏω_vib_ = 0.05 eV) and two electronic potential surfaces (red and blue parabolas, {|*g*⟩, |*e*⟩}). The ground (red parabola) and excited (blue parabola) electronic states are vertically separated by 1.9 eV, and their horizontal shift is denoted by the parameter Δ.

**Figure 1:**
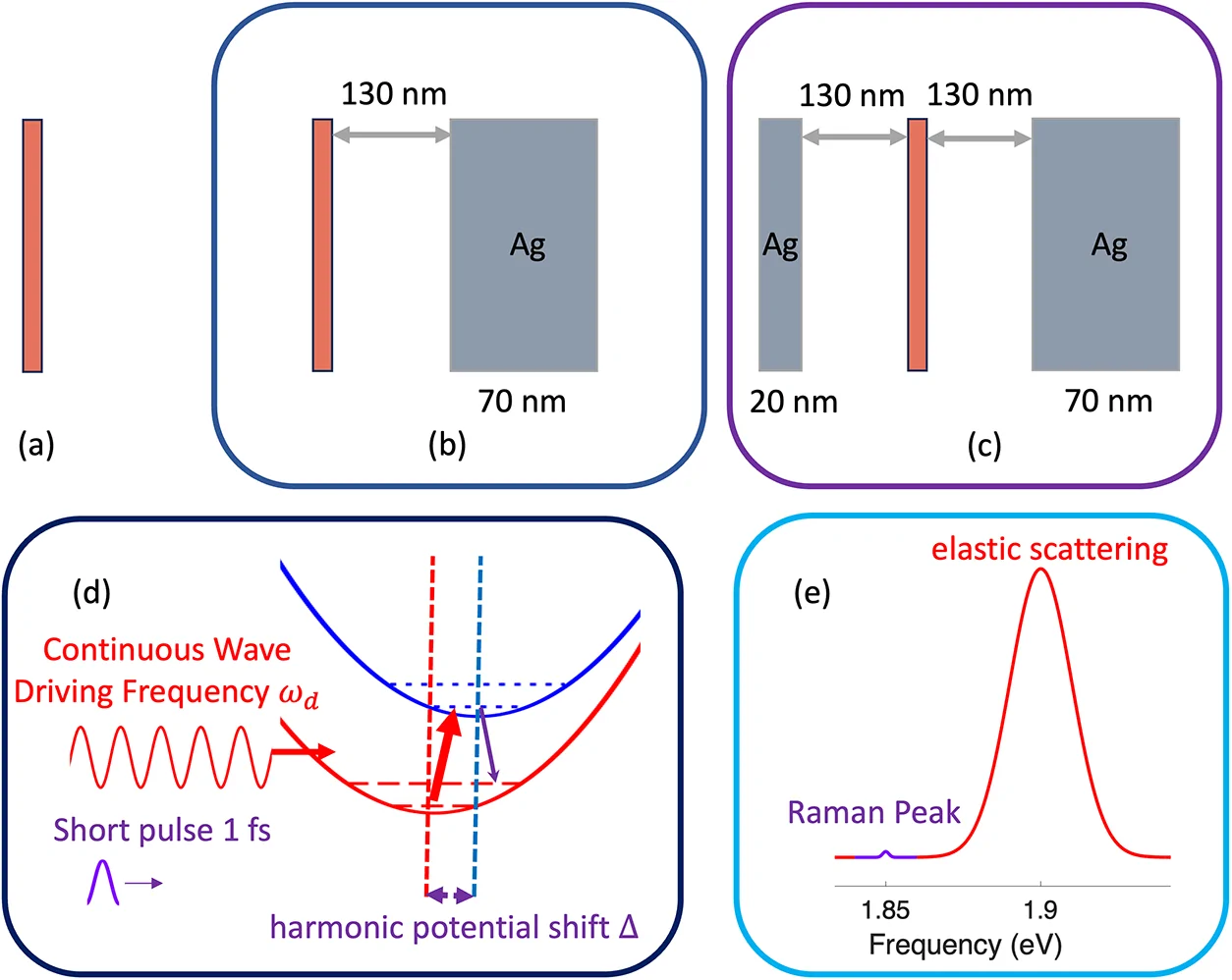
Sketches of three model scenarios: (a) a bare molecular layer, (b) a molecular layer with one 70 nm silver mirror, and (c) a molecular layer inside a cavity formed by 70 + 20 nm mirrors. The 70 nm mirror ensures no transmission leaking to the left side. The 20 nm mirror is thin such that nonresonant light (short pulse and Raman peaks) can efficiently penetrate through. (d) Relevant energy levels inside the molecular layer. The calculation is performed with one continuous wave that promotes population onto the excited electronic state |*e*0⟩ from the ground state |*g*0⟩. After a 3 ps delay, a short pulse stimulates the emission from |*e*0⟩ to |*g*1⟩. (e) Representative output in the absorption spectra subject to the stimulating continuous wave.

The stimulated Raman signal is obtained by (i) driving the system with a continuous wave (red wave) with central frequency ω_*d*_ until a steady state is reached on the electronic excited state (the red arrow) and (ii) upon reaching a steady state, a short pulse (1 fs long) triggers emissions (the purple pulse and arrow). Finally, Figure 1(e) displays a sample absorption spectrum with a bare absorption peak (red) and a small Raman peak (purple). The driving frequency is at ℏω_*d*_ = 1.90 eV, and thus the primary Raman peak appears at ℏω_*d*_ – ℏω_vib_ = 1.85 eV. Note that Figure 1(e) is an ideal schematic figure for purely illustrative purposes.

### II.II Maxwell–Schrodinger Equations

We simulate the electromagnetic field by a set of one-dimensional (1D) Maxwell equations as

1 $$\frac{\partial B_{y}}{\partial t}=-\frac{\partial E_{x}}{\partial z}$$

2 $$\epsilon_{0}\frac{\partial E_{x}}{\partial t}=-\frac{\partial B_{y}}{\mu_{0}\partial z}-J_{x}$$

Here, ϵ_0_ is the vacuum permittivity, μ_0_ is the vacuum permeability, and *z* is the longitudinal coordinate that is perpendicular to the mirror and molecular slab. *J*_*x*_(*z*) is the polarization current on grid point (*z*), defined as

3 $$J_{x}\left(z\right)=\frac{dP_{x}\left(z\right)}{dt}=\varrho \frac{d\left(\langle \psi \left(z,t\right)|{\mathrm{\mu ̂}}_{x}\left(z\right)\left|\psi \left(z,t\right)\right\rangle \right)}{dt}$$

Here, *ϱ* is the number density, μ_*x*_ is a matrix of transition dipole moments between electronic states along the *x* direction.

Within the silver mirror, we define a macroscopic polarization *P*_*x*_ following the standard Drude model

4 $$\frac{\partial^{2}P_{x}}{\partial t^{2}}+\gamma \frac{\partial P_{x}}{\partial t}=\epsilon_{0}\Omega_{p}^{2}E_{x}$$

Here, the damping rate and plasma frequency for silver are defined as γ = 0.243 eV and Ω_*p*_ = 8.90 eV. For details regarding the FDTD solver for Maxwell equations with different dielectric constants, please see refs 22 and 39.

Finally, the evolution of the wave functions |ψ_*z*_*i*__(*r, t*)⟩ inside the molecular layer (|*z*_*i*_| < 10 nm) is described by the Schrodinger equation under a mean-field approximation. In other words, there is one wave function at each spatial grid point within the molecular layer, and the equation of motion is

5 $$i\hbar \frac{d}{dt}\left|\psi_{z_{i}}\left(r,t\right)\right\rangle =Ĥ\left(t\right)\left|\psi_{z_{i}}\left(r,t\right)\right\rangle$$

The time-dependent Hamiltonian at point *z_i_*

$$Ĥ=\left(-\frac{\hbar^{2}}{2m}\frac{\partial^{2}}{\partial r^{2}}+\frac{1}{2}m\omega_{\mathrm{vib}}^{2}{\mathrm{r̂}}^{2}\right)\left|g\right\rangle \left\langle g\right|+\left(-\frac{\hbar^{2}}{2m}\frac{\partial^{2}}{\partial r^{2}}+\frac{1}{2}m\omega_{\mathrm{vib}}^{2}{\left(\mathrm{r̂}-\Delta {Î}_{r}\right)}^{2}+\hbar \omega_{\mathrm{ge}}\right)\left|e\right\rangle \left\langle e\right|-\mu_{x}^{\mathrm{ge}}E_{x}\left(z_{i},t\right)\left(\left|g\right\rangle \langle e\left|+\left|e\right\rangle \langle g\right|\right)$$ 6

includes the local time-dependent electric field as the coupling between adjacent electronic states, i.e., $\left\langle g\right|Ĥ\left|e\right\rangle =\mu_{x}^{\mathrm{ge}}E_{x}\left(z_{i},t\right)$ . Note that because the simulation is one-dimensional, there is no $\mu_{y}^{\mathrm{ge}}E_{y}\left(z_{i},t\right)$ or $\mu_{z}^{\mathrm{ge}}E_{z}\left(z_{i},t\right)$ , and the photonic structures in Figure 1(a–c) represent infinite planes of layers. Here, ω_vib_ = 0.05 eV, Δ is the harmonic potential shift that determines the Franck–Condon spectral structure. All wave functions inside the molecular layer are initialized on the ground vibronic state (|*g*0⟩). Moreover, to ensure the numerical stability of the FDTD calculation, we introduce a phenomenological relaxation time *T*_1_ = 1 ps and a dephasing time *T*_2_ = 10 ps, which is motivated by the inherent lifetime of the excited state. For numerical efficiency, instead of using a set of Liouville equations, we apply a non-Hermitian wave packet approximation as in refs 40,41 , and^42^. The spatial grid in our simulations is a one-dimensional array from −1000 to 1000 nm, with 2000 grid points.^43^ Near the left edge of the simulation cell at *z*_inc_ = −950 nm, we drive one grid point with an electric field that is the sum of a continuous wave and a delayed 1 fs short pulse. This driving generates an electromagnetic (EM) field traveling in both directions. In the rightward direction, the generated EM field reaches the nanolayers at normal incidence. In the leftward direction, the generated EM field is absorbed by a convolutional perfectly matched layer (CPML), a boundary condition that ensures no reflections back into the system.^22^

At each time step, we record the EM fields at the two sides, which are effectively transmission and reflection signals. We perform a Fourier transformation and normalize by the incident amplitudes to find the transmission (**T**) and reflection (**R**) spectra. The absorption spectra are obtained by the normalization condition as **A** = 1 – **T** – **R**.

To obtain the optical response in the presence of an external driving (pump) field, it is necessary to isolate the probe-induced response from the total electromagnetic (EM) field (which contains contributions from both the pump and probe). To this end, we perform two simulations in parallel: one with both pump and probe fields, and one with only the pump field. The probe-induced electric field is then obtained by subtracting the two fields at each time step

E(t;signal)=E(t;pump+probe)−E(t;pumponly) 7

Before the probe pulse is introduced (at *t* = 3 ps), this difference is strictly zero, ensuring that only probe-induced dynamics are captured.

The corresponding Poynting vector is computed as

$$S\left(t;signal\right)=\frac{E\left(t;signal\right)\times B\left(t;signal\right)}{\mu_{0}}$$ 8

Finally, the signal is normalized by the incident probe intensity, and a Fourier transform of the Poynting vector is performed to obtain the absorption spectrum under continuous-wave (CW) pumping. This result is compared with the standard probe-only spectrum (i.e., without pumping) to extract the nonlinear response induced by the pump field. See Appendix B for details of our parameter choice.

## III Results

In this section, we present our FDTD simulation results from two perspectives. First, we present how the linear absorption signal (which in turn determines the Raman signal strength) depends on the harmonic potential shift Δ, because the absorption strength determines the Raman signals strength. Second, we study the stimulated Raman signal strength for varying the driving frequency ω_*d*_ and harmonic potential shift Δ.

### III.I Linear Absorption Intensity for Varying Harmonic Potential Shift

We begin by investigating the linear absorption intensity as a function of the harmonic potential shift Δ between the ground and excited electronic states within the molecular layer. Here, a linear absorption spectrum is obtained by sending a short pulse into the system in the absence of earlier driving. As shown in Figure 2(a), we observe four peaks (within the frequency range) corresponding to transitions from |*g*0⟩ to |*e*0⟩ – |*e*3⟩ with energies 1.90, 1.95, 2.00, and 2.05 eV (within the focused energy range). Moreover, the peaks reach their maxima at Δ ≈ 0, 0.4, 0.58, and 0.71, respectively, in accordance with the maximal overlap between the vibrational wave functions, that is, the Franck–Condon envelope. Next, in Figure 2(b), with a silver mirror placed on the right-hand side of the molecular layer, constructive interference between the incident and reflected fields enhances linear absorption strength (the peaks appear as dark red). Last, in Figure 2(c), the molecular layer is placed between two mirrors with the fundamental cavity model taken in resonance with the molecular |*g*0⟩ → |*e*0⟩ (1.9 eV) transition. We see polaritonic peaks at Δ ≈ 0, and a shrinking of the Rabi splitting as Δ increases and the overlap decreases. Note that the cavity environment further enhances the local field and, consequently, the absorption strength. Note also that the finite quality factor of the silver-mirrored cavity yields very broad polaritonic peaks.

**Figure 2:**
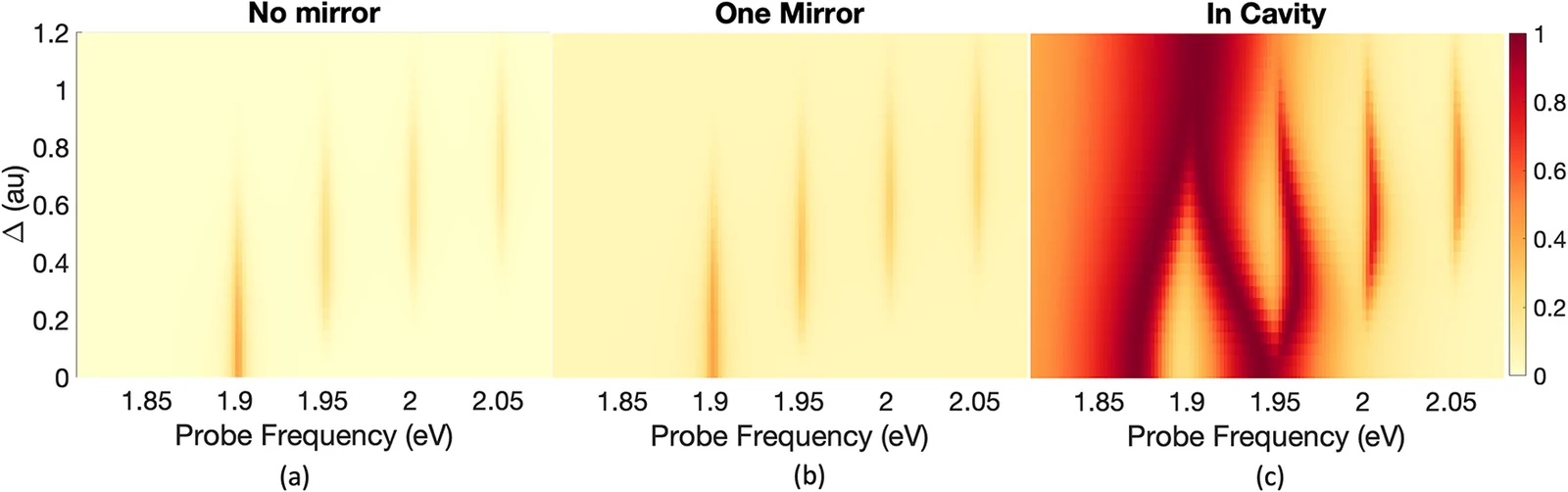
Linear absorption spectra with varying harmonic potential shift Δ for (a) no mirror, (b) one mirror, and (c) inside a cavity. The molecular layer is positioned at a distance of 130 nm from the mirror(s), as indicated in Figure 1. There is relatively smaller absorption for the no mirror scenario because we choose the mirror-molecule distance in scenarios (b, c) such that constructive interference between the incident and reflected waves enhances the EM field amplitude near the molecular layer.

### III.II Raman Intensity

Now, we turn on the driving field with varying central angular frequency ω_*d*_. The integrated stimulated Raman signals (obtained from the difference between the emitted intensity with and without the driving field and integrated over ℏω_out_ = ℏω_*d*_ – ℏω_vib_ ± 0.01 eV) are shown in Figure 3 for different values of ω_*d*_ and Δ for three model systems shown in Figure 1(a)–(c).[Fn fn1]

**Figure 3:**
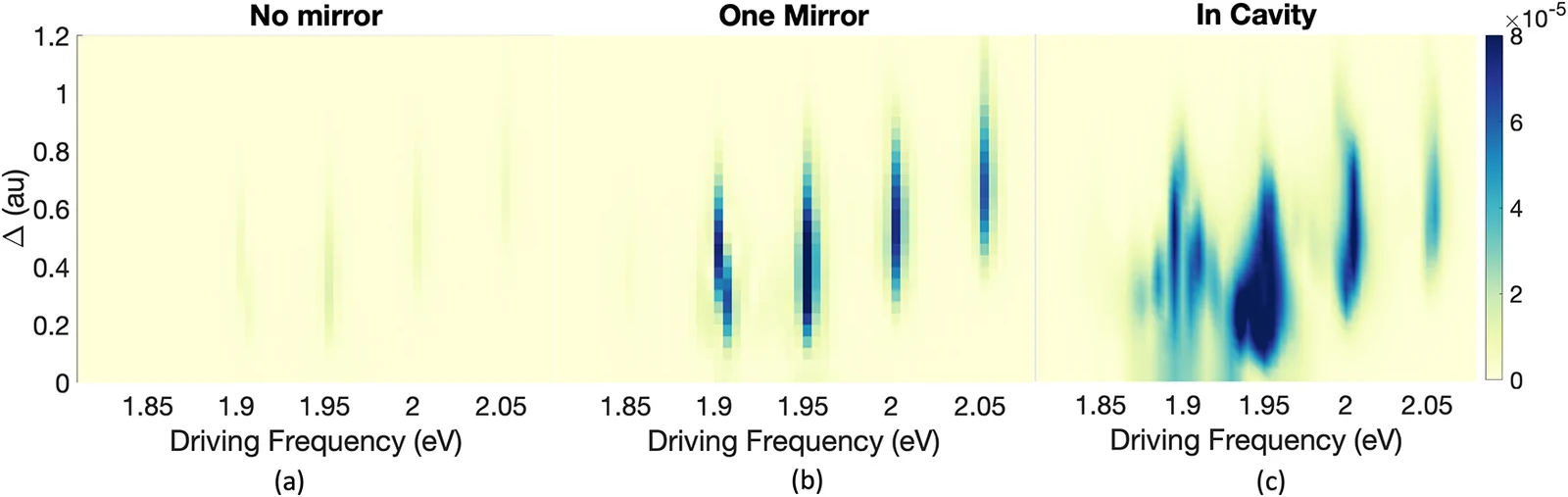
Integrated Raman signal strength for varying driving frequencies (ω_*d*_) and harmonic potentials shift (Δ) for the three scenarios shown in Figure 1(a)–(c). In all cases, the integrated Raman signal strength is found to be proportional to the linear absorption at frequency ω_*d*_ in Figure 2. Inside a cavity, however, the signal has a rich signature resulting from its dependence on the local dielectric response at any given driving frequency ω_*d*_. Thereby, we observe several thin peaks (following the intrinsic long lifetime of the bare molecular excited state |*e*0⟩ and the external pumping field) spanning a large area (following the short lifetime of the polaritonic states).

As shown by comparing Figures 2 and 3(a),(b), the Raman signal intensity increases with the absorption strength, and in fact, we find that these observables are proportional to each other. Furthermore, as expected, the Raman signal intensity vanishes at Δ = 0. Most importantly, as shown in Figure 3(c), a cavity can change some quantitative features of the these Raman signals. First, the broadening due to the cavity in the linear absorption spectra in Figure 2(c) leads to broader Raman signals. Second, when the cavity (model (c)) is driven near the two polariton branches, the Raman signal strength shows an interference pattern that has a structure associated with the stimulating CW line width modulating the polariton signals. In particular, small changes in effective light-matter couplings (associated with their dependence on Δ) and the driving frequencies (ℏω_dr_) lead to phase mismatching and rematching, leading to an oscillatory behavior of the Raman signal strength as a function of the driving frequency ω_*d*_, as seen in Figure 3 (c). Last, we observe a strong signal when the upper polariton coincides with the |*g*0⟩ → |*e*1⟩ transition at 1.95 eV.

One more interesting, and potentially puzzling, observation is the appearance of a Raman signal at Δ = 0 between the two polariton branches at 1.85 and 1.95 eV. Such a signal should vanish in steady-state scattering because, for Δ = 0, there is no coupling between different vibrational states on different surfaces. In a transient pump–probe process, during the time-lag between populating the excited electronic state and the subsequent stimulated emission induced by the short pulse, the depletion of ground state population leads to a Rabi contraction (also seen in Figure 3).^44–47^ In Appendix A, we present the direct comparison between a Rabi contraction signal and a Raman signal.

Next, we investigate how the Raman signal intensity *R* depends on key physical parameters: transition dipole moment μ, molecular number density *n*_0_, and molecular layer thickness *L*_*M*_. To extract the relevant scaling laws, we introduce a common prefactor *C*, apply it to all quantities, and then analyze the Raman signals to extract the corresponding power-law dependencies. As shown in Figure 4(a), in the absence of a cavity, the normalized Raman signal *R* scales linearly with the prefactor *C* for both *n*_0_ and *L*_*M*_. In addition, the Raman signal exhibits the expected μ^4^ dependence, consistent with the two-photon nature of the Raman process.

**Figure 4:**
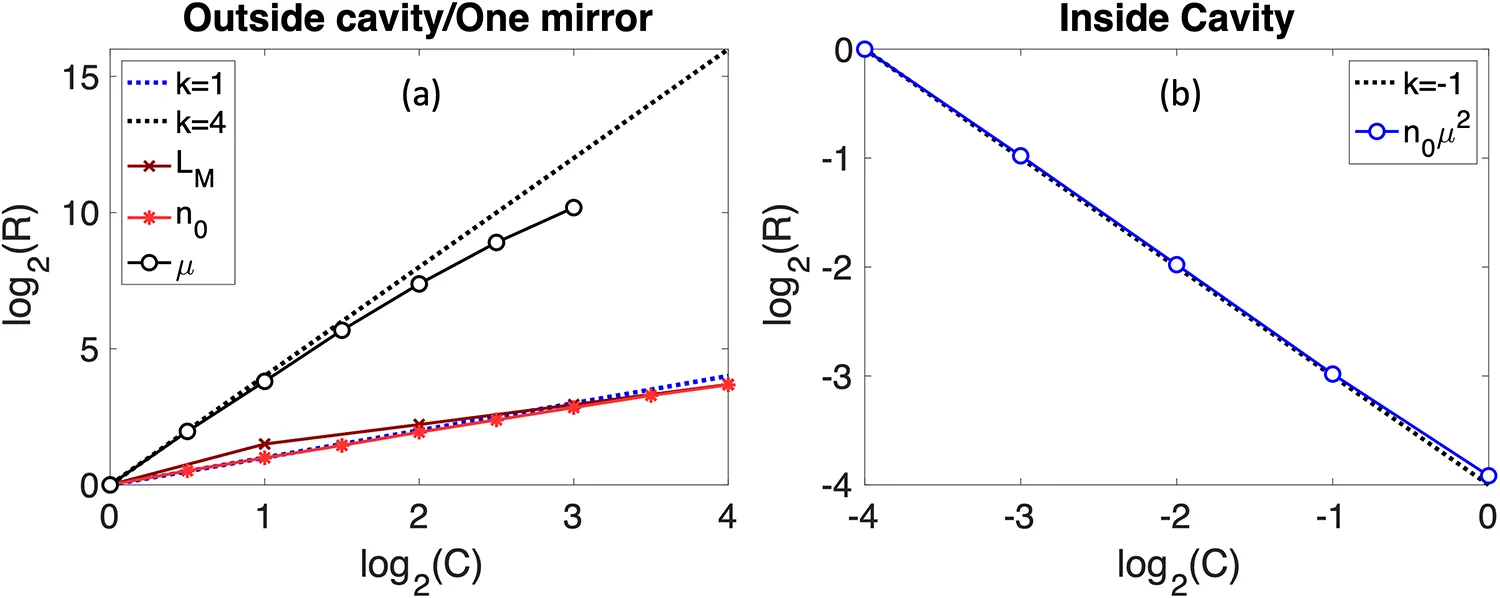
Raman intensity (*R*) dependence on the transition dipole moment μ, molecular density (per unit volume) *n*_0_, and layer thickness *L*_*M*_ for (a) outside a cavity and (b) inside a cavity. On the vertical axis, we plot the log of the Raman peak (nonintegrated) height. The three dotted lines in both subfigures serve as references, corresponding to power-law slopes *k* = 1, *k* = 4, and *k* = −1, respectively. As shown by the red lines in part (a), the Raman intensity scales linearly with both *L*_*M*_ and *n*_0_, indicating the absence of collective effects. Note that *L*_*M*_ and *n*_0_ both characterize the number of molecules in the system and have identical effects on the Raman signals. The black line demonstrates the quartic dependence *R* ∝ μ^4^, consistent with the two-photon nature of the Raman process. For a meaningful quantitative comparison of Raman signals inside the cavity, we impose a constant Rabi splitting, $\Omega_{R}∼\sqrt{\mu^{2}n_{0}L_{M}}\equiv \mathrm{const}$ . Under this constraint, the Raman signal exhibits an apparent inverse dependence on the prefactor, *R* ∝ 1/*C*. See eq 9 for a definition of *C*.

As observed in Figures 2 and 3, the stimulated Raman signal strength is positively correlated with the linear absorption. Therefore, for scenario (c) in Figure 1, in order to isolate cavity-induced effects, we constrain the linear absorption spectrum to remain unchanged while varying system parameters. This is achieved by simultaneously scaling μ and *n*_0_ as

9 $$\mu \to \mu /\sqrt{C},n_{0}\to n_{0}C$$

which keeps the Rabi splitting $\Omega_{R}∼\sqrt{\mu^{2}n_{0}}$ constant.

Under this constraint, Figure 4(b) shows that the Raman signal inside the cavity scales inversely with the prefactor *C* associated with the molecular density *n*_0_. This behavior is fully consistent with the scaling relation *R* ∝ μ^4^ *n*_0_, confirming that the apparent inverse dependence arises from the imposed constraint that preserves the linear absorption (and hence the light–matter coupling strength).

## IV Conclusions

We have investigated the effect on the stimulated Raman signal induced by a single mirror and a low-quality cavity by solving the coupled Maxwell–Schrödinger equations using the FDTD technique. We present the integrated Raman signal as a function of the external driving frequency and the harmonic potential shift. In contrast to a bare molecular layer, we find that, in the single-mirror scenario (b), the enhancement of the Raman signal strength is primarily proportional to the corresponding enhancement in linear absorption induced by the mirror. Furthermore, cavity broadening allows more electromagnetic fields to enter the cavity, leading to stronger Raman peaks near both the lower and upper polariton branches. The interplay between cavity-induced broadening and the external pumping broadening produces detailed features in the Raman signal as a function of the driving frequency ω_*d*_. These features arise from interference of electromagnetic fields within the local dielectric environment, which occurs when the cavity-induced broadening overlaps with the external pumping broadening. At large Rabi splitting, we observe that the upper polariton interferes with a higher vibronic state. All of these phenomena are captured by our FDTD simulation, which explicitly accounts for the full dielectric environment.

Our work highlights the fact that, if we want to quantitatively understand experimental results, it is important to self-consistently treat the molecular quantum subsystem and the local dielectric environment (i.e., a position-dependent macroscopic dielectric function (ϵ­(*z*))). For example, a recent experiment has associated differences in the Raman signal as found in the presence of a cavity environment with a possible change of the Franck–Condon envelope on the molecular potential energy surface.^48^ In the system studied here, we observe a strong cavity effect on a Raman signal that arises not from changes in the molecular potential energy surface but rather from a combination of geometrical and optical factors, a distinction that will need to be investigated further in the future as different effects are teased apart. More generally, looking forward, a subwavelength molecular layer opens many opportunities for engineering optical devices with unique collective responses. Another future direction of the present research is to extend the present framework to describe molecular systems with multiple electronic excited states and intensity borrowing arising from vibronic coupling, a phenomenon that requires more than two electronic levels; non-Condon effects might also be interesting. Finally, it will also be interesting to adopt the present methodology to investigate coherent nonlinear processes such as two-dimensional spectroscopy in the presence of optical nanomaterials, especially in the strong light-matter coupling regime.

## Figures and Tables

**Figure 5:**
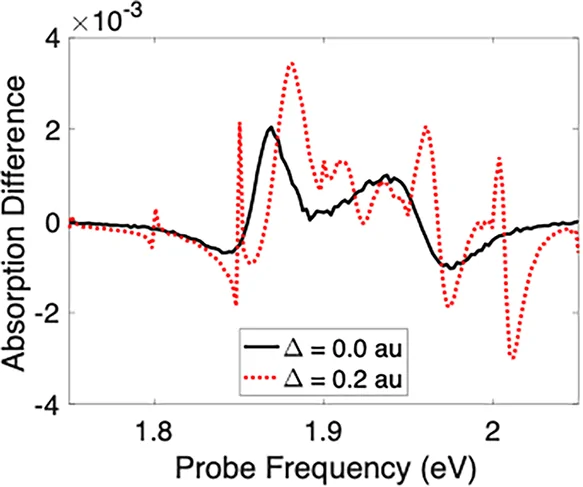
Difference in linear absorption due to external pumping for Δ = 0.0 (black solid line) vs Δ = 0.2 (red dotted line); these signals are second order in the external electric field. For Δ = 0.0, we observe a smooth line with two differential peak structures (corresponding to the lower and upper polariton peaks). For Δ = 0.2, we observe narrower Raman peaks, including the primary one at frequency ℏω = 1.85 eV.

## Footnotes

1 The detailed shapes of these Raman signals as functions of the outgoing frequency are strongly dependent on the local dielectric environment and may not look like a perfect Gaussian–Lorentzian shape as the purple peak in Figure 1­(e).

## Appendix A: Two Competing Second-Order Effects: Rabi Contraction vs Raman Signals

In this appendix, we discuss the nonzero signals found underneath the two polariton branches near Δ ≈ 0 in Figure 3. In Figure 5, the *y*-axis represents the difference in linear absorption for model (c), comparing the case with external continuous-wave pumping (ℏω_dr_ = 1.9 eV) to that without pumping. This procedure is the same as in Figure 3 (effectively removing the linear response associated with the short pulse and isolating the nonlinear contribution), but now we Fourier transform the problem signal and report data as a function of the resulting probe frequency (which should not be confused with the *x*-axis in Figure 3, which is the driving frequency). For the black curve (Δ = 0.0), two differential peaks are observed near the lower and upper polariton energies. Each peak exhibits both negative and positive components, with the positive regions located between the two polariton resonances. These differential peaks indicate that, under pumping, the polariton peaks shift toward each other, a phenomenon known as Rabi contraction.^45,46^ This behavior becomes far more complicated when we introduce a nonzero displacement Δ, and we assign the most prominent Raman peak to be that which occurs at ℏω = 1.85 eV. For this driving frequency, for all values of Δ, we integrate the probe frequency data between 1.84 and 1.86 eV to obtain the data points in Figure 3(c). We have numerically verified that the relative amplitudes of the differential peaks and of the Raman peaks are insensitive to the pumping intensity, suggesting that both contributions are of the same order (though not explicitly shown here).

## Appendix B: Parameters

In this appendix, we summarize all parameters with Table [Table tblI].

**I tblI:** Parameters for Maxwell–Bloch simulation

Name	Value^†^
Molecules’ electronic resonant frequency ℏω_ge_	1.9 eV
Molecules’ resonant wavelength λ_*M*_	652 nm
Molecules vibrational mode frequency ω_vib_	0.05 eV
Molecules’ relaxation time scale *T*_1_	1 ps
Molecules’ dephasing time scale *T*_2_	10 ps
Thickness of the silver mirror	70 and 20 nm
Distance between the silver mirror and the molecular layer	130 nm
Drude model for silver	Ω_*d*_ = 8.90 eV
	Γ_*d*_ = 0.243 eV
Transition dipole moment	μ_*x*_ = 5 D
Simulation grid size (1D)	2000 nm
Number of grid points for EM field	2000
Harmonic potential grid size (1D)	12 au
Number of grid points for the wave function	1200
Number of Wave functions	10
Time step (d*t*)	d*x*/2/*c*_0_
CPML grid points	19
